# Behavioural and Cognitive Changes in Lewy Body Dementias

**DOI:** 10.1155/2018/2404191

**Published:** 2018-11-11

**Authors:** Woon-Man Kung, Ying-Jui Ho, Hiroshi Yoshizawa, Shinro Matsuo, Cheng-Yu Wei

**Affiliations:** ^1^Department of Exercise and Health Promotion, College of Education, Chinese Culture University, Taipei, Taiwan; ^2^Division of Neurosurgery, Department of Surgery, Taipei Tzu Chi Hospital, Buddhist Tzu Chi Medical Foundation, New Taipei City, Taiwan; ^3^Department of Surgery, School of Medicine, Buddhist Tzu Chi University, Hualien, Taiwan; ^4^Department of Psychology, Chung Shan Medical University Hospital, Chung Shan Medical University, Taichung, Taiwan; ^5^Department of Neurology, Neurological Institute, Tokyo Women's Medical University, Kawadacho, Shinjuku, Tokyo, Japan; ^6^Department of Nuclear Medicine, Kanazawa University Hospital, Takaramachi, Kanazawa, Japan; ^7^Department of Neurology, Chang Bing Show Chwan Memorial Hospital, Changhua County, Taiwan

Dementia with Lewy bodies (DLB) and Parkinson's disease dementia (PDD) share many clinical and pathological features. They are therefore discussed together and referred to as Lewy body dementias (LBD).

Incidence of DLB seems low. It is plausible that a systematic review of population and clinical studies is more appropriate to estimate prevalence A recent interesting study addressed the mean prevalence of DLB to be 4.2% and 7.5% in community and healthcare unit, respectively [1]. Another epidemiological article showed the incidence rate of DLB in the United States to be 3.5 per 100,000 person-years but increased steeply to 31.6 per 100,000 person-years among people older than 65 years [2].

After rigorous peer review, we decided to include 10 manuscripts in this special issue, including both research studies and reviews. S.-K. Yang et al. reported the incidence of DLB in Taiwan to be 7.1 per 100,000 person-years while the comorbidity rates of hypertension and hyperlipidemia in DLB patients were higher in females than in males.

Another retrospective longitudinal study conducted by P.-H. Chen et al. implied that 17.2% of patients with mild cognitive impairment progressed to LBD, while older age and higher Clinical Dementia Rating Scale-Sum of Boxes (CDR-SB) were the associated risk factors of progression.

An extensive meta-analysis study conducted by Y.-C. Wang et al. evaluated and clarified the correlation between antidepressants and dementias.

Advanced age, male sex, and a family history of dementia are risk factors of DLB [3]. C.-K. Cheng et al. further discovered evidence demonstrating that metabolic risk factors are important to DLB, and DLB might have higher risk of ischemic stroke, basing on large-scale nationwide, population-based information obtained from the Taiwan National Health Insurance Research Database.

C.-Y. Lee et al. reported the first review article to date focusing on the quality of life in DLB patients.

Psychotic symptoms with recurrent visual hallucination and altered sleep and arousal behaviour are both important features of DLB [4]. In a retrospective registry study, R.-C. Tzeng et al. proposed a high incidence of 51.2% of DLB patients with delusional feature.

P.-C. Chan et al. further did a systemic review on this important issue. They summarized and reviewed the history, clinical manifestations, possible pathophysiologies, and treatment of DLB and REM sleep behaviour disorder (RBD).

Basic science inevitably is the cornerstone of clinical practice. Occipital hypometabolism is a hallmark feature in DLB [5]. By comparing FDG-PET images between DLB patients and healthy subjects, D. Chen et al.'s laboratory investigated brain metabolism patterns in DLB patients.

Treatment of LBD is challenging in everyday practice. Ceftriaxone is a *β*-lactam antibiotic and was suggested to have a potential role in neurodegenerative diseases. By using manganese-enhanced magnetic resonance imaging (MEMRI) and immunohistochemistry, Professor Y.-J. Ho et al. found that ceftriaxone treated DLB rat with improved neuronal density and activity in the hippocampal CA1 area, suppressed hyperactivity in the subthalamic nucleus, and reduced *α*-synuclein accumulation.

*Hericium erinaceus* is an edible mushroom with medicinal and some important physiological components [6]. I.-C. Li et al.'s laboratory reviewed the properties of the mycelia and suggested further research into its therapeutic role.

In this special issue, the authors aim to bring a multidisciplinary perspective and updated insight into the most recent advances in the field of LBD. We hope the efforts should progress our further understanding of the disease.

## References

[1] Vann Jones S. A., O'Brien J. T. (2014). The prevalence and incidence of dementia with Lewy bodies: a systematic review of population and clinical studies. *Psychological Medicine*.

[2] Savica R., Grossardt B. R., Bower J. H., Boeve B. F., Ahlskog J. E., Rocca W. A. (2013). Incidence of dementia with Lewy bodies and Parkinson disease dementia. *JAMA Neurology*.

[3] Nelson P. T., Jicha G. A., Kryscio R. J. (2010). Low sensitivity in clinical diagnoses of dementia with Lewy bodies. *Journal of Neurology*.

[4] Walker Z., Possin K. L., Boeve B. F., Aarsland D. (2015). Lewy body dementias. *The Lancet*.

[5] Filippi M., Agosta F., Barkhof F. (2012). EFNS task force: the use of neuroimaging in the diagnosis of dementia. *European Journal of Neurology*.

[6] Khan M. A., Tania M., Liu R., Rahman M. M. (2013). *Hericium erinaceus*: an edible mushroom with medicinal values. *Journal of Complementary and Integrative Medicine*.

